# Food consumption based on processing level (according to Nova system) during the COVID-19 pandemic among adolescents with immunocompromised conditions: a case-control study

**DOI:** 10.3389/fnut.2023.1141845

**Published:** 2023-05-16

**Authors:** Gabriel P. Esteves, Bruna Caruso Mazzolani, Fabiana Infante Smaira, Heloísa C. Santo André, Amanda Yuri Iraha, Camilla Astley, Isabela Gouveia Marques, Milla Cordeiro Amarante, Nathalia Saffioti Rezende, Sofia Mendes Sieczkowska, Tathiane Christine Franco, Luana Cristina do Amaral Miranda, Lívia Lindoso, Alberto Carame Helito, Jane Oba, Ligia Bruni Queiroz, Rosa Maria R. Pereira, Lucia Maria A. Campos, Nadia E. Aikawa, Hamilton Roschel, Clovis A. Silva, Bruno Gualano

**Affiliations:** ^1^Rheumatology Division, Faculdade de Medicina FMUSP, Applied Physiology and Nutrition Research Group, School of Physical Education and Sport, Universidade de São Paulo, São Paulo, SP, Brazil; ^2^Laboratory of Assessment and Conditioning in Rheumatology, Faculdade de Medicina FMUSP, Disciplina de Reumatologia, Universidade de São Paulo, São Paulo, SP, Brazil; ^3^School of Applied Science (FCA), State University of Campinas, Limeira, SP, Brazil; ^4^Hospital das Clinicas HCFMUSP, Faculdade de Medicina, Instituto da Criança e do Adolescente (ICr), Universidade de São Paulo, São Paulo, Brazil; ^5^Rheumatology Division, Hospital das Clinicas HCFMUSP, Faculdade de Medicina, Universidade de São Paulo, São Paulo, Brazil; ^6^Food Research Center, University of São Paulo, São Paulo, Brazil

**Keywords:** quality of life, quality of sleep, lifestyle, social distancing, youth

## Abstract

The COVID-19 pandemic impacts on eating habits among adolescents may be more relevant in pediatric patients with immunocompromised chronic diseases. This case-control study conducted between June and October 2020 aimed to: (i) describe dietary patterns of adolescents with chronic conditions compared to healthy controls and (ii) determine associations between food consumption, health-related quality of life (HRQL) and sleep quality during the COVID-19 pandemic. Participants (184 immunocompromised and 58 healthy adolescents, aged 14.3 [SD 2.5]) responded to HRQL and sleep validated instruments (PedsQL and PSQI) and three 24 h food recalls via online software. Adjusted linear and logistic regressions were used to assess differences in dietary patterns and associations between food consumption (according to Nova classification) and HRQL and sleep quality. Adolescents with gastrohepatic, rheumatic, and kidney diseases had an improved dietary pattern vs. their healthy peers, showing greater consumption of unprocessed and minimally processed foods (unstandardized coefficient (*b*) = 7.35%[95%CI 1.59; 13.1]; *b* = 15.10%[95%CI 7.00; 23.1]; and *b* = 11.2%[95%CI 5.68; 16.8]), and lower consumption of ultraprocessed foods (*b* = −7.53%[95%CI-12.90; −2.18]; *b* = −11.4%[95%CI-18.90; −3.94]; *b* = −10.8%[95%CI-16.00; −5.68]). Consumption of culinary ingredients was associated with reduced psychological HRQL in controls (standardized coefficient (*β*) = −0.26[95%CI-0.52; −0.004]), and processed food consumption was associated with improved sleep latency in immunocompromised participants (*β* = 0.16[95%CI 0.01; 0.31]). These findings suggest diet quality may play a role in HRQL and sleep quality in this population, and may be relevant for clinical practitioners and policy makers when considering the importance of dietary quality in immunocompromised youths.

## Introduction

Changes in eating habits resulting from the COVID-19 pandemic have been shown worldwide across different ages (1, 2). In healthy adolescents, school closures and home confinement were related to increased unhealthy eating habits, such as eating fried and sweet foods (3). Social distancing measures may be even more restrictive to adolescents with immunocompromised conditions, as they are deemed to be at a higher risk of severe disease (4), and as routine changes, modifications to dietary habits and quality may be even more significant. However, the clinical impact of changes in eating habits during the pandemic is still controversial, as both positive (i.e., participation in home-cooking, eating with others) and negative (i.e., snacking, eating in front of television) changes have been observed (5, 6). Indeed, a previous study from our group showed that adolescents with immunosuppressed chronic conditions changed their eating habits during the COVID-19 pandemic, cooking and consuming more home-made meals, but also eating more in front of the TV (5). These dietary habits may modify diet quality. For instance, home-made cooking may lead to increased consumption of unprocessed and minimally processed foods (UNMP), while eating in front of the TV and screen time may favour ultraprocessed food (UPR) consumption. However, to our knowledge, the diet quality of adolescents with immunocompromised conditions during the pandemic has not been previously investigated.

Conversely, there is growing evidence showing that the COVID-19 pandemic negatively affected health-related quality of life (HRQL) and sleep quality (7, 8). Children and adolescents have also experienced a reduction in their HRQL, likely as a result of the massive changes in their daily lives during the pandemic (9). Moreover, a pronounced shift towards later sleep, combined with an increase in sleep duration, was found in adolescents during the pandemic (10). Diet quality could possibly impact several factors that influence overall HRQL, such as body mass (11), mental health (12), and sleep quality (13). Indeed, a meta-analysis of observational studies showed that UPR food consumption was associated with higher rates of overweight/obesity, and higher risk of depression and cardiovascular events, which can all deteriorate HRQL (14). Regarding sleep quality, healthier diets (i.e., those richer in complex carbohydrates, fiber and unsaturated fats) have been associated with improved directly measured sleep quality (13). In addition, a higher frequency of UPR foods consumption, combined with lower consumption of UNMP foods, was associated with a higher rate of poor sleep quality, both before (15) and during the COVID-19 pandemic (16).

Further investigation is required to better understand the interplay between diet, sleep and HRQL in an underexplored population (youth with pre-existing chronic diseases) during the pandemic. In thesis, potential impairments in diet quality brought about by the pandemic could be particularly harmful for those already suffering from comorbidities. Therefore, the aims of this study were two-fold: (i) to characterize food consumption (according to processing level) in adolescents with chronic conditions vs. healthy controls during the COVID-19 quarantine and (ii) to investigate possible associations between food consumption, HRQL and sleep quality parameters in adolescents with immunocompromised chronic conditions and healthy controls.

## Methods

### Study design and setting

This report is part of a larger observational and exploratory study aimed at screening lifestyle and overall health in youth with multiple chronic conditions; this cross-sectional, case-control assessment was conducted between July and October 2020, during which important social distancing measures were in place to contain the spread of COVID-19 in São Paulo, Brazil (5, 17).

### Participants

Participants were recruited from the Children’s Institute of the Clinics Hospital of the University of São Paulo, the largest tertiary, referral, teaching hospital in Latin America. A total of 512 adolescents with chronic conditions (aged between 10 and 18 years) were invited to participate in this study, with 184 having accepted to participate in the study and fulfilling questionnaires and dietary food recalls. Participants recruitment was done by outpatient doctors that provided full explanation of study design and aims to adolescents and legal guardians. An informed consent form was signed digitally by both adolescents and legal guardians before beginning the survey.

The initial sample of 512 adolescents comprised a variety of immunocompromised conditions: juvenile rheumatic diseases [juvenile dermatomyositis (*n* = 23), juvenile idiopathic arthritis (*n* = 83), childhood-onset systemic lupus erythematosus (*n* = 43)], gastrointestinal and hepatic conditions [celiac disease (*n* = 12), eosinophilic esophagitis (*n* = 23), inflammatory bowel disease (*n* = 44), autoimmune hepatitis (*n* = 28), and liver transplant (*n* = 50)], and kidney conditions [nephrotic syndrome (*n* = 22), chronic kidney disease (*n* = 7), and kidney transplant (*n* = 12)]. Given the wide variety of clinical conditions, we grouped adolescents with immunocompromised chronic conditions in 3 overarching disease categories: gastrohepatic, rheumatic and kidney diseases. Additionally, 126 healthy adolescents, frequency-matched by age and sex, were recruited through social media and local newspapers to serve as controls, with 58 meeting the eligibility criteria (i.e., absence of pre-existing chronic conditions) and adequately fulfilling the survey.

### Variables and data measurement

All participants were instructed on how to adequately complete at home a comprehensive self-reported online survey through the Research Electronic Data Capture® (REDCap®) platform, and were urged to ask for assistance if they had difficulty in answering any questions at home. This online-survey included: (i) demographic characteristics (i.e., age, sex, ethnicity, and educational level), (ii) three 24 h food recall, filled in non-consecutive days (two of them during weekdays and one during the weekend), in which participants were instructed to fully report the quantity and preparation of foods and beverages they consumed within the previous 24 h, with an exemplary and adequately completed 24 h recall made available for consultation, and (iii) validated instruments to assess HRQL and sleep quality, namely the Pediatric Quality of Life Inventory (PedsQL) (18) and the Pittsburg Sleep Quality Index (PSQI) (19) score.

The PedsQL contains 23-items comprising four domains: physical (8 items), emotional (5 items), social (5 items) and school (5 items) functioning (18). Each item is scored on a 3 or 5-point scale and scores are linearly transformed to a 0–100 scale, with higher scores relating to improved HRQL (18). PedsQL was assessed with the appropriate forms considering children different ages of developing 8–12, and 13–18 years. The PSQI consists of 19-self-rated questions comprising the following components: subjective sleep quality, sleep latency, sleep duration, habitual sleep efficiency, sleep disturbances, use of sleeping medication and daytime dysfunction (19). The global score ranges from 0–21 and scores higher than 5 indicates bad sleep quality (19). Further information on how each of the metrics related to the PSQI are scored can be found elsewhere (20). Both these questionnaires were previously validated for Portuguese, displaying adequate reliability coefficients (18, 19).

Food recalls were preliminary evaluated by the research staff, and when information was insufficient or inaccurate (e.g., size of food portions not reported), participants were re-contacted so that the necessary information could be obtained. Then, food intake analysis was performed using Dietbox online software (Dietbox.me, Rio Grande do Sul, BR), with food preparations (e.g., soups, puree, pies, and sandwiches) being broken down into individual food ingredients according to standardized recipes. Total energy intake (kcal) and macronutrient intake [total grams and percentage of total energy intake (%TEI)] were calculated. The contribution of each food processing category according to the Nova classification system [i.e., UNMP, processed culinary ingredients (PCI), processed (PR) and UPR] was assessed through adequate classification of individual foods (see the Supplementary Material for an overview of each food processing category and examples according to Monteiro et al. (21)), and by calculating each food processing category’s weight contribution relative to total daily food intake (%Weight). The approach of using weight, as opposed to caloric contribution (%TEI), was used as it better captures the influence of certain ultraprocessed foods that have low caloric content, but might still contribute significantly to a given dietary pattern (e.g., zero-energy, artificially sweetened beverages (22)). A separate, sensitivity analysis using caloric contribution (%TEI) was also done, and is available as Supplementary Tables S1, S2. Finally, patients’ disease status was assessed through medical records. The study was approved by the Research Ethics Committee of Clinical Hospital (Approval Number: 31314220.5.0000.0068).

### Study size

Sample size was determined considering: (a) the number of potentially eligible patients from our outpatient services, (b) the staffing capacity (technicians, assistants, students, and researchers) of our research team, and (c) the availability of resources available to conduct the project (23, 24). We simulated the minimum effect size necessary to reach 80% power given our sample size and study design. Using G*Power software (version 3.1.9.7) (25), and considering the small to moderate effect size of Cohen’s *f*^2^ = 0.06, an alpha of 0.05, and 7 predictors in the linear multivariable regression model (2 continuous, 3 categorical, with 2 added dummy variables for education status), we found that a total sample size of 247 would have resulted in 80% statistical power.

### Statistical methods

Descriptive data are presented as mean (SD) for continuous variables and as absolute and relative frequencies [*n* (%)] for categorical variables. The association between disease status and food processing level was assessed using linear regression, with disease status (control, and rheumatic, gastrohepatic, and kidney diseases) considered as independent variables, and with multiple models used, one for each food processing category (%Weight of UNMP, PCI, PR, and UPR foods). Additionally, the association between food processing level and quality of life and sleep quality was also assessed. For quality of life, variables obtained from PedsQL were the physical, psychological and overall HRQL scores. For sleep quality, variables of interest obtained from the PSQI were the classification of sleep quality using the global sleep score (“good quality” for scores ≤ 5, and “bad quality” for scores > 5 (16)), “Sleep Latency” and “Sleep Efficiency.” Sleep latency scores were inverted, so that higher values would represent a positive outcome. Associations were tested using logistic regression for binary dependent variables (i.e., classification of sleep quality as “good” or “bad”), and linear regression for continuous dependent variables, with the various outcomes obtained from the questionnaires considered as dependent variables, and food processing level considered as independent variables. To assess possible differences between healthy controls and immunocompromised patients, these associations were tested in all participants, and then in healthy controls and immunocompromised patients, separately. All models were adjusted for age, sex, self-reported ethnicity and current education status. An alpha of 0.05 was previously set for all analyses.

Association results are presented as unstandardized coefficients (*b*) and their respective 95% confidence intervals. For the association between food processing and HRQL and sleep quality variables, beta coefficients are presented in standardized form (*β*), so that variables of different magnitudes could be appropriately compared. All models were checked for underlying assumptions of regression, such as normality and linearity of residual values, using appropriate graphical visualizations. All analyses were done using R (26) (version 4.2.2) and Rstudio (2022.02.3, PBC, USA), with the *dplyr* (27) and *ggplot2* (28) packages used for data wrangling and plotting, and the R core package *stats* (26) for statistical models.

## Results

In total, 242 (184 immunocompromised patients and 58 healthy controls) participants provided adequate 24 h dietary recall and questionnaire data (Table 1). Mean age of participants was 14.2 (2.57), 13.6 (2.43), 14.7 (2.37), and 14.5 (2.43) for healthy controls, gastrohepatic, rheumatic, and kidney disease groups. Most participants were males in the healthy control, gastrohepatic, and rheumatic disease groups, but more females were present in the kidney disease group. Most adolescents were at elementary or high school level.

**Table 1:** Description of participants’ characteristics, food consumption, health-related quality of life, and sleep.

	Healthy controls (*n* = 58)	Gastrohepatic disease (*n* = 86)	Kidney disease (*n* = 24)	Rheumatic disease (*n* = 74)
Age, years	14.2 (2.57)	13.6 (2.43)	14.5 (2.43)	14.7 (2.37)
Sex				
Male	39 (67%)	49 (57%)	7 (29%)	56 (75%)
Female	19 (33%)	37 (43%)	17 (71%)	18 (25%)
Ethnicity				
Caucasian	36 (62%)	51 (59%)	13 (54%)	35 (47%)
Non-Caucasian	22 (38%)	35 (41%)	11 (46%)	39 (53%)
Current education status				
Not studying	4 (7%)	1 (1%)	0 (0%)	2 (3%)
Elementary school	34 (58%)	56 (66%)	14 (59%)	36 (48%)
High school	17 (29%)	27 (31%)	9 (37%)	30 (41%)
University	3 (6%)	2 (2%)	1 (4%)	6 (8%)
Food consumption				
Total energy intake (kcal)	1910.52 (700.54)	1880.22 (598.57)	1572.36 (492.80)	1774.46 (536.67)
Carbohydrate intake (%VET)	46.80 (7.45)	49.73 (5.79)	49.33 (6.12)	46.50 (7.54)
Protein intake (%VET)	16.72 (4.53)	16.25 (3.53)	17.19 (2.86)	17.70 (4.97)
Lipids intake (%VET)	36.93 (5.50)	34.25 (4.79)	33.65 (4.45)	35.94 (5.26)
UNMP foods (%Weight)	53.04 (16.79)	63.90 (15.45)	67.88 (15.44)	60.12 (17.20)
PCI (%Weight)	3.01 (1.42)	2.97 (1.44)	3.35 (1.17)	3.10 (1.29)
PR foods (%Weight)	12.37 (9.35)	11.86 (8.18)	8.69 (7.90)	12.59 (9.38)
UPR foods (%Weight)	31.57 (17.0)	21.24 (13.93)	20.05 (15.38)	24.16 (15.21)
Sleep quality				
Sleep latency	1.53 (1.04)	1.01 (0.97)	1.00 (1.08)	1.23 (1.05)
Sleep efficiency	0.68 (0.80)	0.46 (0.86)	0.66 (0.90)	0.63 (1.00)
Sleep quality classification				
Good (<5 score)	18 (40%)	42 (54%)	12 (67%)	38 (52%)
Bad (>5 score)	26 (60%)	35 (46%)	6 (33%)	34 (48%)
Health-related quality of life				
Physical health	7.10 (4.54)	8.20 (6.54)	6.54 (3.80)	9.29 (6.54)
Psychological health	18.91 (7.33)	19.80 (9.73)	15.90 (8.30)	19.68 (8.18)
Total score	26.01 (10.29)	28.00 (14.97)	22.33 (10.61)	28.98 (13.18)

UNMP, unprocessed and minimally processed; PCI, processed culinary ingredients; PR, processed; UPR, ultraprocessed. Data presented as mean (SD) for continuous numerical variables and absolute and relative frequency [*n* (%)] for categorical variables.

**Figure 1:**
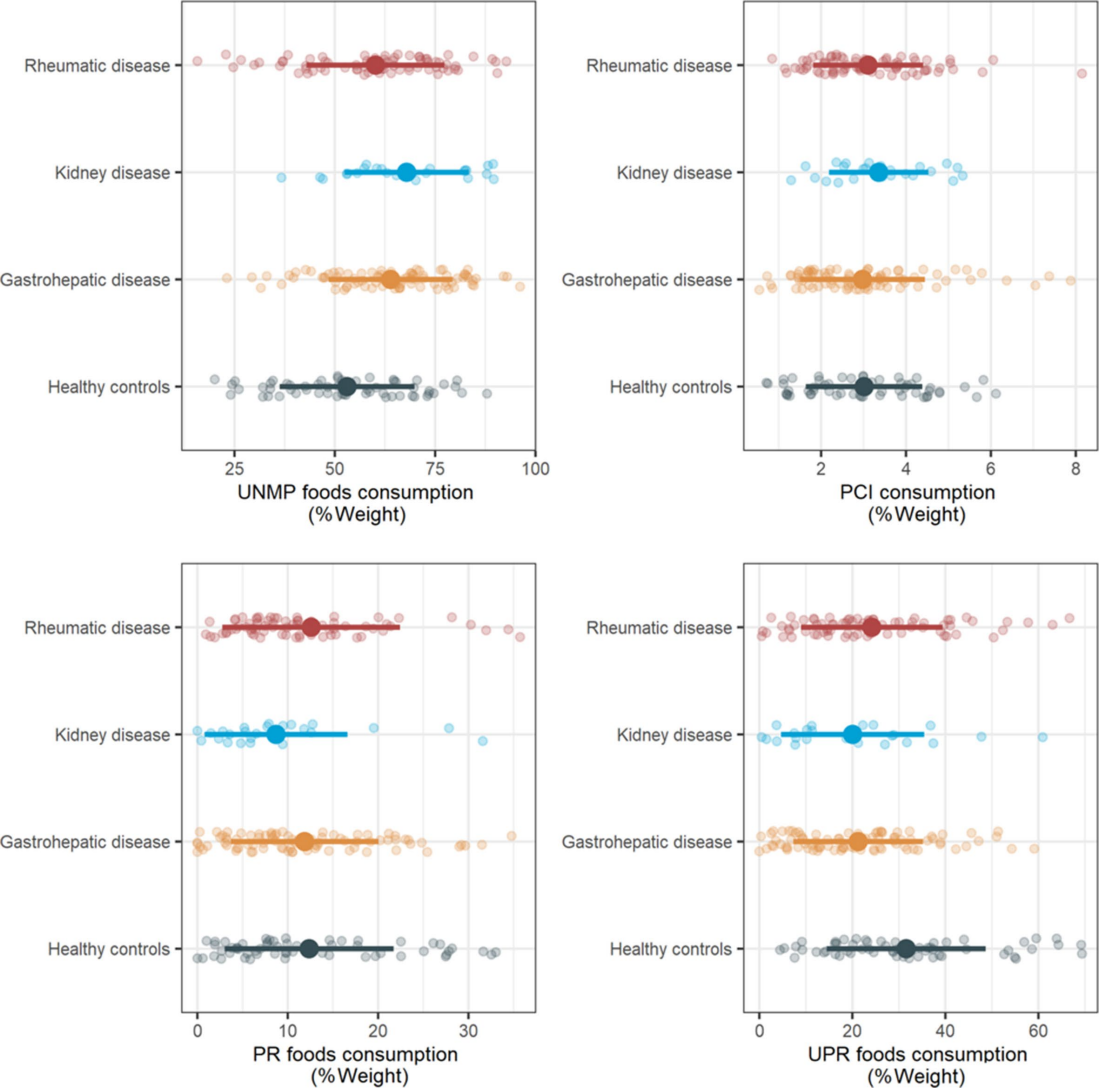
Food processing level consumption across healthy controls, rheumatic, gastrohepatic and kidney disease groups. UNMP, unprocessed and minimally processed; PCI, processed culinary ingredients; PR, processed; UPR, ultraprocessed. Data are presented as mean (SD), alongside individual datapoints.

**Figure 2:**
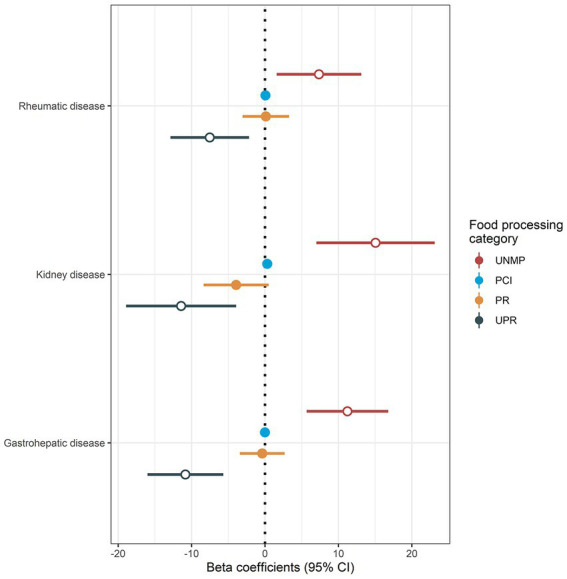
Association between chronic conditions and food processing level consumption. UNMP, unprocessed and minimally processed; PCI, processed culinary ingredients; PR, processed; UPR, ultraprocessed; CI, confidence interval. Results are presented as beta coefficients alongside 95% confidence intervals. Open circles denote statistical significance (*p* < 0.05). Reference values (intercept) were obtained from healthy controls (not shown). Disease status groups (healthy controls, rheumatic, kidney, and gastrohepatic diseases) were considered as independent variables, and consumption of different food processing categories were considered as dependent variables, with separate models used for each food processing category. Models were adjusted for age, sex, self-reported ethnicity, and current education status.

Consumption of the different food processing categories across groups is shown in Figure 1. While PCI and PR foods consumption appeared to be similar between groups, UNMP foods consumption was higher in all disease groups compared to healthy controls. On the other hand, UPR foods consumption was lower in all disease groups compared to healthy controls. A *post-hoc* analysis did not show any differences on food consumption between high schoolers vs. elementary school children or Caucasian vs. non-Caucasians (data not shown). Indeed, adjusted linear regression models showed an association between rheumatic, kidney and gastrohepatic disease groups, and increased UNMP foods consumption (*b* = 7.35% [95% CI 1.59; 13.1], *p* = 0.0125; *b* = 15.10% [95% CI 7.00; 23.1], *p* < 0.001; and *b* = 11.2% [95% CI 5.68; 16.8], *p* < 0.001, respectively), and decreased UPR foods consumption (*b* = −7.53% [95% CI−12.90; −2.18], *p* < 0.01; *b* = −11.4% [95% CI-18.90; −3.94], *p* < 0.01, *b* = −10.8% [95% CI-16.00; −5.68], *p* < 0.001, respectively), relative to healthy controls (Figure 2). There was no association between disease groups and PCI and PR consumption (see Supplementary Table S1 for a summary of all models).

**Figure 3:**
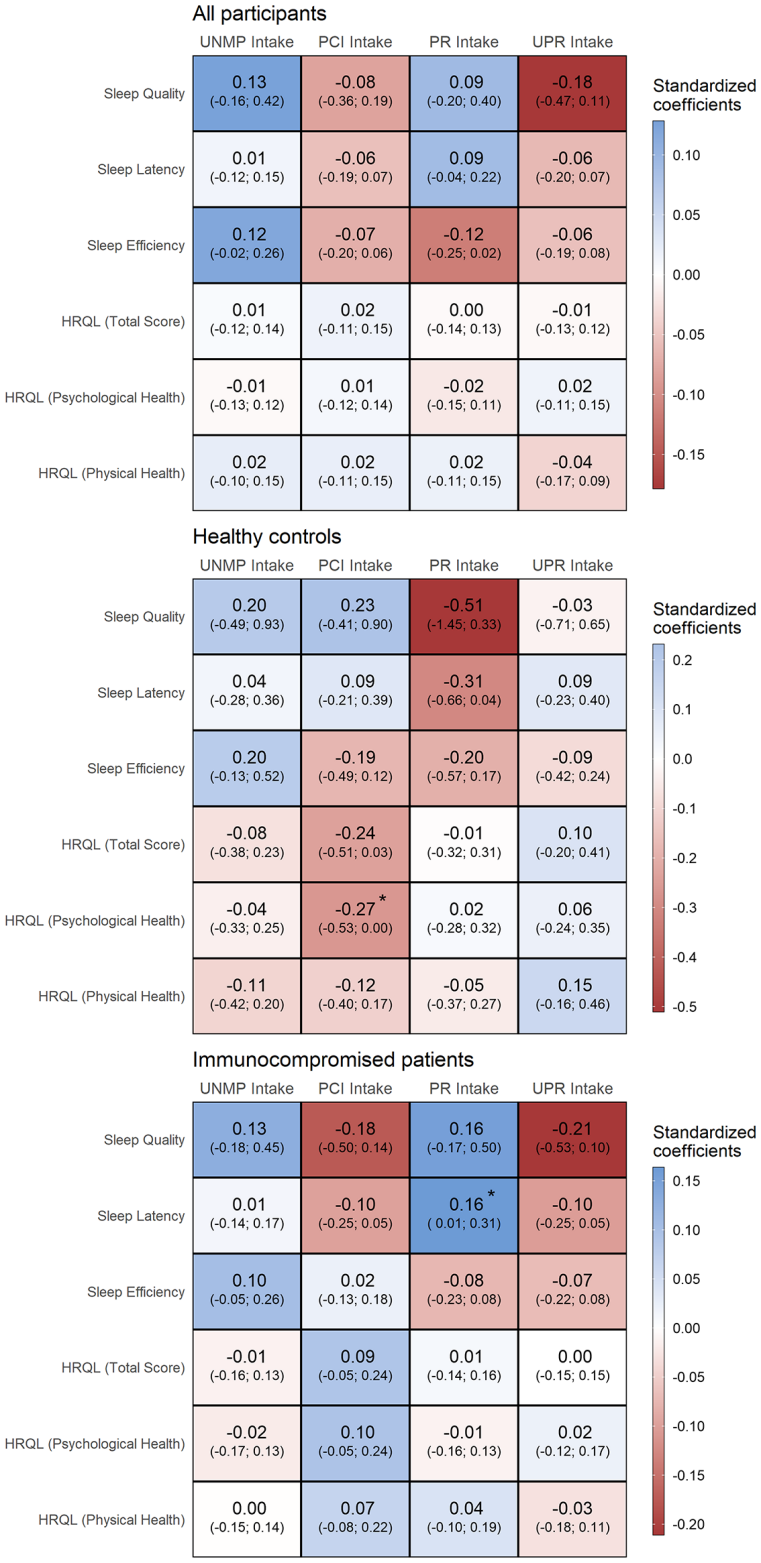
Associations between food processing level consumption and quality of life and sleep quality in all participants, healthy controls and immunocompromised participants. UNMP, unprocessed and minimally processed; PCI, processed culinary ingredients; PR, processed; UPR, ultraprocessed; HRQL, health-related quality of life. * denotes statistical significance (*p* < 0.05). Consumption of food processing categories were considered as independent variables, and quality of life and quality of sleep outcomes from questionnaires were considered as dependent variables. Models were adjusted for age, sex, self-reported ethnicity, and current education status.

Results from adjusted logistic and linear regression models investigating the association between food processing consumption and HRQL and sleep quality parameters are presented in Figure 3. When analysing the entire sample, no significant associations were found (all *p* > 0.05, with confidence intervals crossing 0 for all associations). When healthy controls and immunocompromised were analysed separately, an association between PCI consumption and worsened psychological health scores was found exclusively in the healthy controls group (*β* = −0.26 [95% CI-0.52; −0.004], value of *p* = 0.047), and an association between PR foods consumption and improved sleep latency was found exclusively in the immunocompromised group (*β* = 0.16 [95% CI 0.01; 0.31], value of *p* = 0.032) (Figure 3).

## Discussion

To our knowledge, this is the first study to characterize and compare food consumption of adolescents with immunocompromised chronic conditions to healthy controls during the COVID-19 pandemic. The main findings were: (a) adolescents with immunocompromised chronic conditions showed a higher consumption of UNMP and a lower consumption of UPR foods compared to healthy controls and (b) food consumption by processing level was not associated with HRQL and sleep quality parameters across the entire sample, but PCI consumption was associated with a worsened psychological HRQL score in the healthy control group, and PR foods consumption was associated with improved sleep latency in the immunocompromised group.

In the last decade, the consumption of UPR foods has increased in Brazil (29, 30) and in other countries (31, 32), regardless of age and socioeconomic status. During the COVID-19 pandemic, adolescents reported an increase in screen time and changes in eating habits (3, 5), such as eating more frequently in front of the TV and snacking, habits that were associated with increased consumption of UPR foods during the pandemic in the general population (33). This can be worrisome for adolescents with chronic conditions, since increases in UPR food consumption have been associated with a worse prognosis of the underlying disease and increased risk of kidney function decline in the general population (34), and increased risk of cardiovascular (35) and inflammatory bowel disease (36) in adults with clinical conditions. In the current study, however, adolescents with chronic conditions showed an improved dietary pattern compared to healthy peers. Different reasons can explain this finding. Basing the diet on UNMP foods has been routinely recommended by researchers and clinicians as an adjuvant strategy to manage comorbidities (37–39), which could have favoured patients’ adoption of a healthier eating pattern; parents of adolescents with chronic conditions may be more mindful of nutrition and dietary habits, and thus may seek the adoption of these habits in their household; and finally, other changes in eating habits that have happened during the pandemic, such as increasing the habit of cooking and preparing home-made meals have been observed in this cohort (5), which could result in an increase consumption of UNMP instead of UPR foods. However, these hypotheses cannot be confirmed by the current work. Additionally, since we did not have pre-pandemic data, we cannot determine whether or to which extent food consumption changed as a function of the pandemic.

Sleep quality and overall quality of life are important topics for all individuals with immunocompromising diseases, but especially for adolescents. Adequate sleep is essential for adolescents, with current guidelines from the National Sleep Foundation recommending 8–10 h of sleep a night for this group (40). Food consumption may influence HRQL by modifying factors related to overall health, such as quality of sleep (13–15, 41). A higher consumption of UPR foods (i.e., soft drinks and sweets) has been associated with worse sleep quality (15, 41), while UNMP food consumption has been associated with improvements in sleep (41). More broadly, food consumption may influence HRQL due to their impacts on the prevalence of non-communicable chronic diseases and cardiovascular conditions (14). In the present study, an association between consumption of PCI and worsened psychological HRQL was seen in healthy controls, but not in immunocompromised participants. The excessive consumption of added culinary ingredients, such as salt and sugar, might be related to unhealthy eating behaviours, which may favour a worsened HRQL (42). Regarding sleep, while no associations between food consumption and sleep quality were seen in the healthy controls group, a small, but statistically significant association between PR foods consumption and improved sleep latency (i.e., less time necessary to fall asleep) was found in the immunocompromised group.

PR foods are foods that have their shelf-life increased through the combination of unprocessed foods and culinary ingredients. In the Brazilian diet, PR foods are mostly represented by bakery goods, such as bread, and processed dairy products, such as cheeses (43). This finding is in some ways consistent with other data showing that a high-carbohydrate, low-fat diet leads to a 5 min reduction (and therefore, an improvement) in sleep latency in adult participants (13, 44). Another explanation is that participants that consume more PR products may consume less UPR products, and this sort of dietary pattern might be beneficial for sleep latency. It is unclear why this association was not present in the healthy controls group, although this could be related to either the previous finding that the overall dietary pattern was improved in patients vs. healthy peers, or to an insufficient power among the controls.

Although other associations were not statistically significant, visualizing the point estimate would suggest a positive association between sleep quality and UNMP foods consumption, and a negative association with UPR foods consumption, which aligns with previous studies; however, imprecision was too large to statistically confirm these observations. Thus, we cannot rule out a type 2 error in this study, meaning that a relationship between the variables might exist, but the current sample size was insufficient to identify it. Another possibility is that other factors beyond dietary ones, such as socioeconomical status or mental health, might have been more strongly associated with HRQL and sleep quality during the pandemic in this population.

The strength of this study is that it provides unique data on diet and its association with well-being in a cohort of adolescents with immunosuppressed chronic conditions during the COVID-19 pandemic. Limitations of the study include its observational design, which do not allow comparing food consumption before and during the pandemic, as well as inferring causality. Also, the use of a self-reported questionnaire and 24 h food recalls are prone to knowing reported biases (45), which may have introduced imprecision in the data. We also did not evaluate disease flare scores and disease complications in each chronic condition, since these adolescents were quarantined during the present study due to COVID-19 pandemic. Due to restrictions imposed by the pandemic, we were unable to collect anthropometric and physical activity measures. This could be recognized as a limitation since these parameters could associate with food consumption, HRQL and sleep quality. Additionally, although results of this report cannot be generalized to other populations, inferences specifically guided towards the specific populations analysed herein can be drawn.

In conclusion, adolescents with immunocompromised chronic conditions showed increased consumption of UNMP foods and decreased consumption of UPR foods compared to healthy ones during the COVID-19 pandemic. Moreover, although food consumption was not associated with HRQL and sleep quality parameters across the whole sample, a negative association between PCI consumption and psychological HRQL was seen in healthy controls, and a positive association between PR foods consumption and sleep latency was seen in immunocompromised participants, suggesting food processing might impact these variables in the context of the pandemic. These results reinforce that both adolescents with immunocompromised conditions and healthy ones should reduce UPR food consumption, either by increasing UNMP or PR food consumption, and that PCI should be used discretely, preferably during the preparation of home-cooked meals. Further studies assessing the lifestyle changes introduced by the COVID-19 pandemic and their potential impact on overall health and well-being in this vulnerable population remain warranted.

## Data availability statement

The raw data supporting the conclusions of this article will be made available by the authors, without undue reservation.

## Ethics statement

The studies involving human participants were reviewed and approved by Research Ethics Committee of the São Paulo Clinical Hospital (HCFMUSP). Written informed consent to participate in this study was provided by the participants’ legal guardian/next of kin.

## Author contributions

GE, BM, FS, and HS: responsible for study design, collection, analysis, and interpretation of data, the writing of the report, and the decision to submit the manuscript for publication. AI, CA, IM, MA, NR, SS, TF, LA, LL, AH, JO, LQ, LC, and NA: responsible for study design, the collection of data, reviewing drafts of the article, and approval of the final version. RP, HS, CS, and BG: responsible for analysis, interpretation of data, the writing of the report, and the decision to submit the manuscript for publication. All authors contributed to the article and approved the submitted version.

## Funding

This work was supported by São Paulo Research Foundation—FAPESP (grants #2020/02741-1, #2020/07860-9, #2021/02742-0, #2015/26937-4, #2019/14820-6, #2017/13552-2, #2015/03756-4, #2019/14819-8, #2019/20814-9, #2019/15231-4, #2016/00006-7); the National Council for Scientific and Technological Development (CNPq, #304984/2020-5; #305556/2017-7); and the Núcleo de Apoio à Pesquisa “Saúde da Criança e do Adolescente” da USP (NAP–CriAd).

## Conflict of interest

The authors declare that the research was conducted in the absence of any commercial or financial relationships that could be construed as a potential conflict of interest.

## Publisher’s note

All claims expressed in this article are solely those of the authors and do not necessarily represent those of their affiliated organizations, or those of the publisher, the editors and the reviewers. Any product that may be evaluated in this article, or claim that may be made by its manufacturer, is not guaranteed or endorsed by the publisher.
