# Supplementary material for: Food consumption based on processing level (according to Nova system) during the COVID-19 pandemic among adolescents with immunocompromised conditions: a case-control study
Source: Front Nutr. 2023 May 16;10:1141845. doi: 10.3389/fnut.2023.1141845 (PMC10227604; doi:10.3389/fnut.2023.1141845)
Supplement: Supplementary file 1 [file Data_Sheet_1.docx]

**Supplementary Table 1.** Estimate results from models comparing food consumption across immunocompromised patients and healthy controls

| **Term** | **β coefficient (95% CI)** |  |  |  |
| --- | --- | --- | --- | --- |
| *Food processing and chronic diseases (%Weight) ^a^* | UNMP (%Weight) | PCI (%Weight) | PR (%Weight) | UPR (%Weight) |
| Healthy controls | 0 [Reference] | 0 [Reference] | 0 [Reference] | 0 [Reference] |
| Rheumatic disease | 7.35 (1.59; 13.11) ^b^ | 0.06 (-0.42; 0.54) | 0.11 (-3.05; 3.28) | -7.53 (-12.88; -2.18) ^c^ |
| Kidney disease | 15.05 (7; 23.11) ^d^ | 0.31 (-0.37; 0.98) | -3.93 (-8.36; 0.49) | -11.42 (-18.91; -3.94) ^c^ |
| Gastrohepatic disease | 11.23 (5.68; 16.78) ^d^ | -0.01 (-0.47; 0.46) | -0.38 (-3.43; 2.68) | -10.84 (-16; -5.68) ^d^ |
| *Food processing and chronic diseases (%TEI) ^a^* | UNMP (%TEI) | PCI (%TEI) | PR (%TEI) | UPR (%TEI) |
| Healthy controls | 0 [Reference] | 0 [Reference] | 0 [Reference] | 0 [Reference] |
| Rheumatic disease | 5.53 (0.98; 10.08) ^b^ | 0.68 (-1.21; 2.56) | -0.31 (-4.5; 3.87) | -5.81 (-10.4; -1.22) ^b^ |
| Kidney disease | 10.67 (4.3; 17.03) ^c^ | 3.41 (0.77; 6.05) ^b^ | -3.37 (-9.23; 2.48) | -10.82 (-17.24; -4.4) ^c^ |
| Gastrohepatic disease | 7.26 (2.87; 11.64) ^c^ | 0.28 (-1.53; 2.1) | 0.11 (-3.92; 4.15) | -7.33 (-11.75; -2.9) ^c^ |

Table shows results from the main models using the relative weight contribution (%Weight) of each food consumption category, which is presented in the paper, and also using the relative energetic contribution (% total caloric intake, TEI) as the dependent variable.

^a^ = Disease status groups (healthy controls, rheumatic, kidney and gastrohepatic diseases) were considered as independent variables, and consumption of different food processing categories were considered as dependent variables (either %Weight or %TEI). Models were adjusted for age, sex, self-reported ethnicity and current education status.

^b^ = p<0.05; ^c^ = p<0.01; ^d^ = p<0.001.

UNMP = unprocessed and minimally processed; PCI = processed culinary ingredients; PR = processed; UPR = ultraprocessed; TEI = total energy intake.

**Supplementary Table 2.** Estimate results from quality of life, sleep quality and food processing models

| **Term** | **β coefficient (95% CI)** |  |  |  |  |  |
| --- | --- | --- | --- | --- | --- | --- |
| *Health-related quality of life, sleep quality and food processing (%Weight) ^a^* | Sleep quality | Sleep latency | Sleep efficiency | HRQL (Total Score) | HRQL (Physical Health) | HRQL (Psychological Health) |
| **All participants** |  |  |  |  |  |  |
| UNMP (%Weight) | 0.18 (-0.1; 0.46) | 0.05 (-0.08; 0.18) | 0.1 (-0.03; 0.24) | 0.01 (-0.12; 0.14) | 0.02 (-0.1; 0.15) | -0.01 (-0.13; 0.12) |
| PCI (%Weight) | -0.09 (-0.36; 0.18) | -0.06 (-0.19; 0.07) | -0.07 (-0.2; 0.06) | 0.02 (-0.11; 0.15) | 0.02 (-0.11; 0.15) | 0.01 (-0.12; 0.14) |
| PR (%Weight) | 0.09 (-0.2; 0.39) | 0.1 (-0.04; 0.23) | -0.12 (-0.25; 0.02) | 0 (-0.14; 0.13) | 0.02 (-0.11; 0.15) | -0.02 (-0.15; 0.11) |
| UPR (%Weight) | -0.23 (-0.51; 0.05) | -0.1 (-0.23; 0.03) | -0.04 (-0.17; 0.09) | -0.01 (-0.13; 0.12) | -0.04 (-0.17; 0.09) | 0.02 (-0.11; 0.15) |
| **Healthy controls** |  |  |  |  |  |  |
| UNMP (%Weight) | 0.2 (-0.49; 0.93) | 0.04 (-0.28; 0.36) | 0.2 (-0.13; 0.52) | -0.08 (-0.38; 0.23) | -0.11 (-0.42; 0.2) | -0.04 (-0.33; 0.25) |
| PCI (%Weight) | 0.23 (-0.41; 0.9) | 0.09 (-0.21; 0.39) | -0.19 (-0.49; 0.12) | -0.24 (-0.51; 0.03) | -0.12 (-0.4; 0.17) | -0.27 (-0.53; 0) ^b^ |
| PR (%Weight) | -0.51 (-1.45; 0.33) | -0.31 (-0.66; 0.04) | -0.2 (-0.57; 0.17) | -0.01 (-0.32; 0.31) | -0.05 (-0.37; 0.27) | 0.02 (-0.28; 0.32) |
| UPR (%Weight) | -0.03 (-0.71; 0.65) | 0.09 (-0.23; 0.4) | -0.09 (-0.42; 0.24) | 0.1 (-0.2; 0.41) | 0.15 (-0.16; 0.46) | 0.06 (-0.24; 0.35) |
| **Immunocompromised patients** |  |  |  |  |  |  |
| UNMP (%Weight) | 0.13 (-0.18; 0.45) | 0.01 (-0.14; 0.17) | 0.1 (-0.05; 0.26) | -0.01 (-0.16; 0.13) | 0 (-0.15; 0.14) | -0.02 (-0.17; 0.13) |
| PCI (%Weight) | -0.18 (-0.5; 0.14) | -0.1 (-0.25; 0.05) | 0.02 (-0.13; 0.18) | 0.09 (-0.05; 0.24) | 0.07 (-0.08; 0.22) | 0.1 (-0.05; 0.24) |
| PR (%Weight) | 0.16 (-0.17; 0.5) | 0.16 (0.01; 0.31) ^b^ | -0.08 (-0.23; 0.08) | 0.01 (-0.14; 0.16) | 0.04 (-0.1; 0.19) | -0.01 (-0.16; 0.13) |
| UPR (%Weight) | -0.21 (-0.53; 0.1) | -0.1 (-0.25; 0.05) | -0.07 (-0.22; 0.08) | 0 (-0.15; 0.15) | -0.03 (-0.18; 0.11) | 0.02 (-0.12; 0.17) |
| *Health-related quality of life, sleep quality and food processing (%TEI) ^a^* | Sleep quality | Sleep latency | Sleep efficiency | HRQL (Total Score) | HRQL (Physical Health) | HRQL (Psychological Health) |
| **All participants** |  |  |  |  |  |  |
| UNMP (%TEI) | 0.22 (-0.06; 0.51) | 0 (-0.13; 0.14) | 0.1 (-0.03; 0.24) | -0.01 (-0.14; 0.12) | 0.02 (-0.11; 0.15) | -0.03 (-0.16; 0.1) |
| PCI (%TEI) | 0.02 (-0.26; 0.3) | 0.03 (-0.1; 0.17) | -0.04 (-0.18; 0.1) | 0.03 (-0.1; 0.16) | 0.03 (-0.09; 0.16) | 0.02 (-0.12; 0.15) |
| PR (%TEI) | 0.01 (-0.28; 0.31) | 0.06 (-0.07; 0.2) | -0.09 (-0.23; 0.05) | 0.03 (-0.1; 0.16) | 0.04 (-0.09; 0.17) | 0.02 (-0.11; 0.15) |
| UPR (%TEI) | -0.26 (-0.55; 0.02) | -0.1 (-0.23; 0.04) | 0 (-0.14; 0.13) | 0 (-0.13; 0.12) | -0.05 (-0.18; 0.08) | 0.03 (-0.1; 0.16) |
| **Healthy controls** |  |  |  |  |  |  |
| UNMP (%TEI) | 0.36 (-0.45; 1.22) | 0.07 (-0.3; 0.43) | 0.21 (-0.17; 0.58) | -0.17 (-0.49; 0.15) | -0.22 (-0.54; 0.1) | -0.1 (-0.41; 0.21) |
| PCI (%TEI) | 0.65 (-0.07; 1.5) | 0.22 (-0.09; 0.52) | -0.13 (-0.45; 0.19) | -0.26 (-0.53; 0.01) | -0.2 (-0.49; 0.08) | -0.24 (-0.51; 0.03) |
| PR (%TEI) | -0.66 (-1.62; 0.19) | -0.35 (-0.71; 0.01) | -0.2 (-0.59; 0.18) | 0.2 (-0.1; 0.51) | 0.09 (-0.23; 0.4) | 0.23 (-0.06; 0.53) |
| UPR (%TEI) | -0.1 (-0.77; 0.57) | 0.09 (-0.22; 0.4) | 0.05 (-0.27; 0.37) | 0.09 (-0.21; 0.38) | 0.2 (-0.1; 0.49) | 0 (-0.29; 0.28) |
| **Immunocompromised patients** |  |  |  |  |  |  |
| UNMP (%TEI) | 0.18 (-0.13; 0.51) | -0.04 (-0.19; 0.11) | 0.1 (-0.05; 0.26) | -0.01 (-0.16; 0.14) | 0.01 (-0.14; 0.16) | -0.03 (-0.17; 0.12) |
| PCI (%TEI) | -0.14 (-0.46; 0.17) | -0.03 (-0.18; 0.12) | 0.03 (-0.12; 0.18) | 0.1 (-0.04; 0.25) | 0.09 (-0.06; 0.24) | 0.1 (-0.05; 0.24) |
| PR (%TEI) | 0.1 (-0.22; 0.43) | 0.13 (-0.02; 0.28) | -0.05 (-0.2; 0.1) | 0.01 (-0.14; 0.16) | 0.05 (-0.1; 0.19) | -0.02 (-0.17; 0.13) |
| UPR (%TEI) | -0.26 (-0.59; 0.06) | -0.1 (-0.25; 0.05) | -0.06 (-0.21; 0.09) | -0.01 (-0.15; 0.14) | -0.07 (-0.21; 0.08) | 0.04 (-0.11; 0.19) |

Table shows results from the main models using the relative weight contribution (%Weight) of each food consumption category, which is presented in the paper, and also using the relative energetic contribution (% total caloric intake, TEI) as the dependent variable.

^a^ = Consumption of food processing categories were considered as independent variables, and quality of life and quality of sleep outcomes from questionnaires were considered as dependent variables. Models were adjusted for age, sex, self-reported ethnicity and current education status.

^b^ = p<0.05.

UNMP = unprocessed and minimally processed; PCI = processed culinary ingredients; PR = processed; UPR = ultraprocessed; TEI = total energy intake; HRQL = health-related quality of life.

**NOVA classification**

In the current study, food processing levels were classified as follows: Group 1 – Unprocessed or minimally processed foods, which include plants or animals after separation from nature and natural foods altered by the processes designed to preserve natural foods, to make them suitable for storage, safe, and edible or more pleasant to consume, such as drying, fractioning, and filtering (e.g., seeds, fruits, roots, milk). Group 2 – Culinary ingredients, which are substances derived from Group 1 or from nature by processes that include pressing, refining, grinding, milling, and drying (e.g., oils, sugar, and salt). Group 3 – Processed foods, which are made essentially by adding salt, oil, sugar, and other substances or foods from Groups 1 and 2 (e.g., homemade breads, cheeses, dried meats). Group 4 – Ultra-processed foods, which are formulations made mostly or entirely from substances derived from foods and additives, with little, if any, intact food from Group 1 (e.g., sausages, candies, snacks). For a more comprehensive explanation on the NOVA system and an extensive list of examples, please refer to the supplemental material in Monteiro et al (2019) (1).

**Reference:**

1. Monteiro CA, Cannon G, Levy RB, Moubarac J-C, Louzada ML, Rauber F, Khandpur N, Cediel G, Neri D, Martinez-Steele E, et al. Ultra-processed foods: what they are and how to identify them. *Public Health Nutr* (2019) 22:936–941. doi: 10.1017/S1368980018003762
